# Tobacco Smoking During Pregnancy Is Associated With Increased Risk of Moderate/Severe Bronchopulmonary Dysplasia: A Systematic Review and Meta-Analysis

**DOI:** 10.3389/fped.2020.00160

**Published:** 2020-04-28

**Authors:** Gema E. González-Luis, Elke van Westering-Kroon, Eduardo Villamor-Martinez, Maurice J. Huizing, Mohammed A. Kilani, Boris W. Kramer, Eduardo Villamor

**Affiliations:** ^1^Department of Pediatrics, Hospital Universitario Materno-Infantil de Canarias, Las Palmas de Gran Canaria, Spain; ^2^Department of Pediatrics, School for Oncology and Developmental Biology (GROW), Maastricht University Medical Center (MUMC+), Maastricht, Netherlands

**Keywords:** maternal smoking, bronchopulmonary dysplasia, very preterm infants, systematic review, meta-analysis

## Abstract

Epidemiological evidence and animal studies support that intrauterine exposure to tobacco smoke disturbs lung development and has a negative effect in the pulmonary health of the offspring. Individual studies suggest an association between fetal exposure to maternal smoking and risk of developing bronchopulmonary dysplasia (BPD). However, this association has not yet been systematically investigated. We aimed to conduct a systematic review of studies reporting on tobacco smoking during pregnancy as potential risk factor for BPD. PubMed/MEDLINE and EMBASE databases were searched. BPD was defined as requirement of supplemental oxygen on postnatal day 28 (BPD28; all BPD), at the postmenstrual age (PMA) of 36 weeks (BPD36; moderate/severe BPD), or as requirement of more than 30% oxygen and/or positive pressure at 36 weeks PMA (severe BPD). Pooled risk ratios (RR) and 95% confidence intervals (CI) were calculated using a random-effects model. Of 2,894 potentially relevant studies, 33 met the inclusion criteria. The included studies evaluated 171,772 infants and included 30,445 cases of exposure to maternal smoking and 25,340 cases of BPD of any severity. Meta-analysis showed a significant association between tobacco smoking during pregnancy and BPD36 (17 studies, RR 1.126, 95% CI 1.008–1.259, *p* = 0.036), but could not demonstrate a significant association between tobacco smoking during pregnancy and BPD28 (16 studies, RR 1.021, 95% CI 0.924–1.129, *p* = 0.681), or severe BPD (3 studies, RR 1.143, 95% CI 0.528–2.478, *p* = 0.734). In conclusion, our data suggest that tobacco smoking during pregnancy increases the risk of moderate/severe BPD. Our results highlight the detrimental effects of tobacco smoking and reinforce the hypothesis of the involvement of prenatal insults in the etiopathogenesis of BPD.

## Introduction

Bronchopulmonary dysplasia (BPD), the chronic lung disease of prematurity, is the most common complication of very and extreme preterm birth (1–6). In addition to low gestational age (GA) and postnatal determinants of lung injury, experimental and clinical data support the role of adverse antenatal factors, such as chorioamnionitis, gestational hypertensive disorders, gestational diabetes, or maternal obesity, in the pathogenesis of BPD (3–5, 7–9). In fact, it has been suggested that prenatal insults may be sufficient to disrupt pulmonary development and to induce lung structural changes, even in the absence of additional postnatal stress (5). Alternatively, exposure to an adverse intrauterine environment may alter susceptibility to critical postnatal factors, leading to altered risk of BPD (5).

Tobacco smoking during pregnancy is associated with a range of detrimental effects in offspring, including stillbirth, congenital anomalies, intrauterine growth restriction, preterm birth, and neonatal mortality (10–14). Epidemiological evidence and animal studies support that intrauterine exposure to tobacco smoke disturbs lung development and has a negative effect in the pulmonary health of the offspring (10–12). Moreover, recent population-based studies suggest an association between fetal exposure to maternal smoking and risk of developing BPD (5, 7, 15, 16). However, to the best of our knowledge, the evidence on the association between intrauterine exposure to tobacco smoke and BPD has not yet been systematically evaluated. Therefore, our objective was to conduct a systematic review and meta-analysis of the observational studies reporting data on the association between maternal smoking during pregnancy and risk of BPD.

## Methods

We followed the same methodology that was used in our previous meta-analysis on the association between chorioamnionitis and BPD (9). The Population, Exposure, Comparison, Outcome (PECO) question was: Do preterm infants (P) exposed to maternal tobacco smoking during pregnancy (E) have a higher risk of developing BPD (O) than preterm infants with no history of exposure to maternal tobacco smoking during pregnancy (C)? A protocol was developed prospectively that detailed the specific objectives, criteria for study selection and inclusion, assessment of study quality, relevant clinical outcomes, and statistical methodology. The review protocol was not previously published or registered, but is available upon request. With the exception of the lack of protocol registration, the study was performed and reported according to the guidelines of the Preferred Reporting Items for Systematic Reviews and Meta-Analysis (PRISMA) (17).

### Sources

A comprehensive literature search was undertaken using the PubMed/MEDLINE and EMBASE databases from their inception to March 1, 2019. The search terms involved various combinations of the following keywords: “maternal smoking,” “bronchopulmonary dysplasia,” “chronic lung disease,” “preterm birth,” “prematurity,” “low birth weight,” “very low birth weight,” “observational study,” “cohort study,” “case-control,” and “risk factors.” Search strategy is detailed in Supplementary Material.

### Study Selection

Two investigators (MJH and EV) independently evaluated studies for inclusion and any disagreements were resolved by discussion. Studies were included for analysis if satisfying all following criteria: (1) full text available in English, German, Dutch, Spanish, French, Italian, Portuguese, or Catalan; (2) with a prospective or retrospective cohort study or case-control design; (3) assessing BPD (any definition, except the combined outcome death or BPD) as outcome of infants exposed to tobacco smoking during pregnancy (self-reported or assessed through laboratory tests), or assessing the risk factors for BPD, when tobacco smoking during pregnancy was one of the investigated risk factors; and (4) in a study population of newborn infants with a gestational age below 34 weeks or birth weight below 1,500 g. Studies not complying with the above-mentioned criteria were excluded. We did not set an a priori minimum sample size of the studies to be included.

### Data Extraction, Assessment of Study Quality, and Assessment of Risk of Bias

Two investigators (GG-L and EW-K) independently extracted data, and two reviewers (EV-M and EV) checked the data extraction for completeness and accuracy. Data extracted from each study included citation information, language of publication, location where research was conducted, time period of the study, study objectives, study design, definitions of maternal smoking and BPD, inclusion/exclusion criteria, patient characteristics, and results (including raw numbers, summary statistics, and adjusted analyses on maternal smoking and BPD where available). Criteria for classification of BPD were similar to those used in our previous meta-analysis (9): “BPD defined as supplemental oxygen requirement on postnatal day 28 was coded as BPD28. BPD defined as oxygen requirement at the postmenstrual age (PMA) of 36 weeks (with or without physiologic challenge of supplemental oxygen withdrawal) was coded as BPD36. Using these definition criteria, BPD28 was considered to include all severities of BPD, whereas BPD36 was considered to include a combination of moderate and severe BPD (9, 18). Finally, severe BPD was defined as need for? 30% oxygen and/or positive pressure at 36 weeks PMA.”

Methodological quality was assessed using the Newcastle-Ottawa Scale (NOS) for cohort studies (19). This scale uses a star rating system (range: 0–9 stars) scoring three aspects of the study: selection (0–4), comparability (0–2), and outcomes (0–3). Risk of bias (RoB) was assessed for each study with the instrument developed by Morgan et al. for non-randomized studies dealing with effects of environmental exposures on health outcomes (20). This tool is modeled on the RoB In Non-randomized Studies of Interventions (ROBINS-I) instrument and takes into consideration seven RoB items: “(1) Bias due to confounding, (2) Bias in selection of participants into the study, (3) Bias in classification of exposures, (4) Bias due to departures from intended exposures, (5) Bias due to missing data, (6) Bias in measurement of outcomes, and (7) Bias in selection of reported results. Judgments for each RoB item can be: Low, Moderate, Serious, or Critical. Similarly, an overall judgment about the bias at the study level is either Low, Moderate, Serious, or Critical RoB” (20). Finally, following the approach proposed by Morgan et al. (20), we integrated the RoB assessments within the Grading of Recommendations Assessment, Development, and Evaluation (GRADE) framework to assess the certainty of the evidence in the systematic review.

### Statistical Analysis

Methods for statistical analysis were identical to those used in our previous meta-analysis on choriamnionitis and BPD (9) and are therefore literally reproduced here. Studies were combined and analyzed using comprehensive meta-analysis V3.0 software (Biostat Inc., Englewood, NJ, USA). The Mantel-Haenszel risk ratio (RR) with 95% confidence interval (CI) was calculated from the raw data provided in the studies. When raw data were not available, published RRs, or odds ratios (ORs) were extracted. ORs were converted to risk ratios following the method of Zhang et al. (21). Due to anticipated heterogeneity, summary statistics were calculated with a random-effects model. This model accounts for variability between studies, as well as within studies. Statistical heterogeneity was assessed by Cochran's *Q* statistic and by the *I*^2^ statistic, which is derived from *Q* and describes the proportion of total variation that is due to heterogeneity beyond chance (22). The *I*^2^ statistic was interpreted as per Higgins & Green (23): low (25% ≤ *I*^2^ < 50%), moderate (50% ≤ *I*^2^ < 75%), and high (*I*^2^ ≥ 75%). Sources of heterogeneity were explored through subgroup analyses and random effects (method of moments) meta-regression analyses (24). The following potential sources of heterogeneity were explored: mean or median GA of the included infants, mean or median birth weight (BW) of included infants, rate of maternal smoking in the total group, design of the study (cohort of case-control), sample size of the study, and continent. Subgroup analysis was performed on continent (America or Europe), design (cohort or case-control), and sample size (higher or lower than 100). We used the Egger's regression test and funnel plots to assess publication bias. A probability value of <0.05 (0.10 for heterogeneity) was considered statistically significant.

## Results

### Description of Studies, Assessment of Quality, and RoB

**Figure 1:**
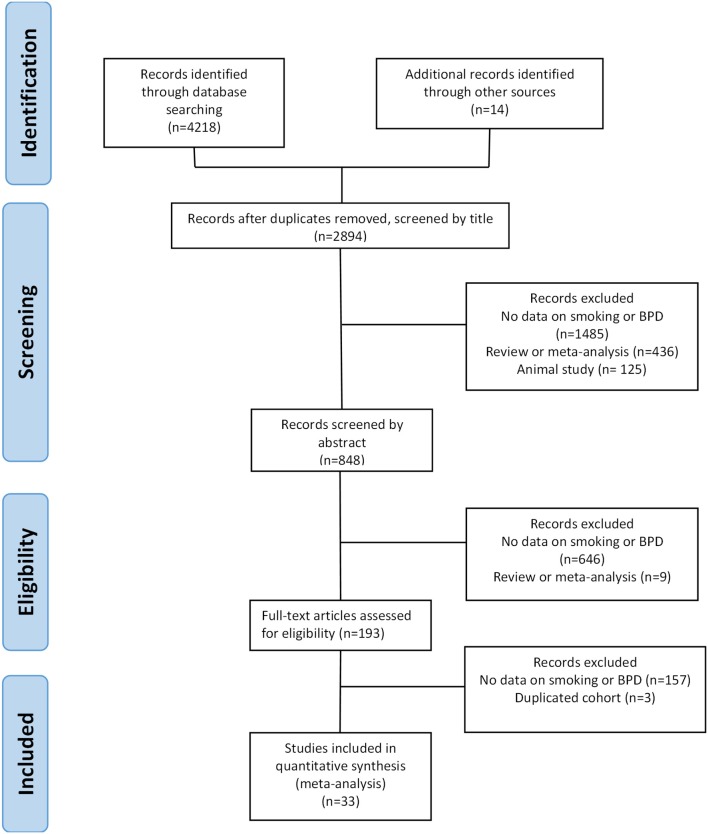
PRISMA diagram of the systematic search.

Of 2,894 potentially relevant studies, 33 (7, 8, 15, 16, 25–53) met the inclusion criteria. The PRISMA flow diagram of the search process is shown in Figure 1. The included studies evaluated 171,772 infants and included 30,445 cases of exposure to maternal smoking and 25,340 cases of BPD of any severity. The included studies and their characteristics are summarized in Supplementary Table 1.

Each study was allocated >6 NOS stars. RoB of the 33 included studies was rated as moderate in the domains of confounding, and classification of exposures (20). Overall RoB of the 33 included studies was rated as moderate.

### Meta-Analysis

**Figure 2:**
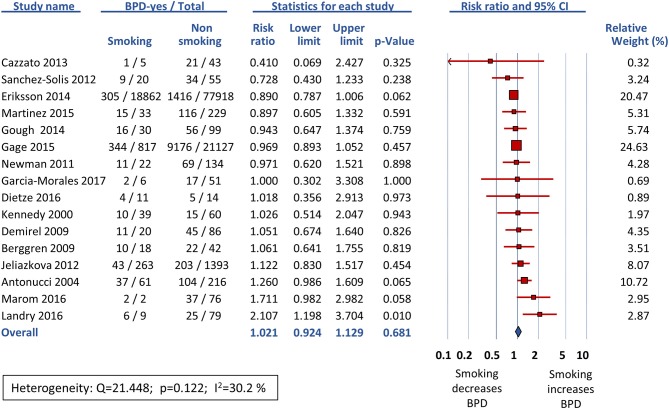
Random effects meta-analysis of the association between maternal smoking during pregnancy and BPD defined as oxygen requirement on postnatal day 28 (BPD28).

**Figure 3:**
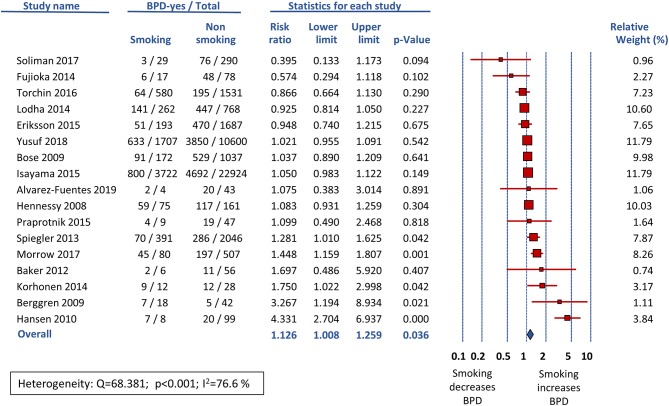
Random effects meta-analysis of the association between maternal smoking during pregnancy and BPD defined as oxygen requirement at the postmenstrual age of 36 weeks (BPD36).

**Figure 4:**
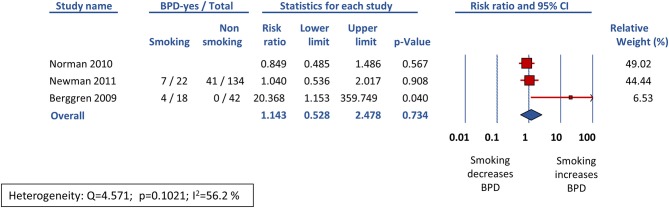
Random effects meta-analysis of the association between maternal smoking during pregnancy and severe bronchopulmonary dysplasia (defined as need for > 30% oxygen and/or positive pressure at 36 weeks postmenstrual age).

**Figure 5:**
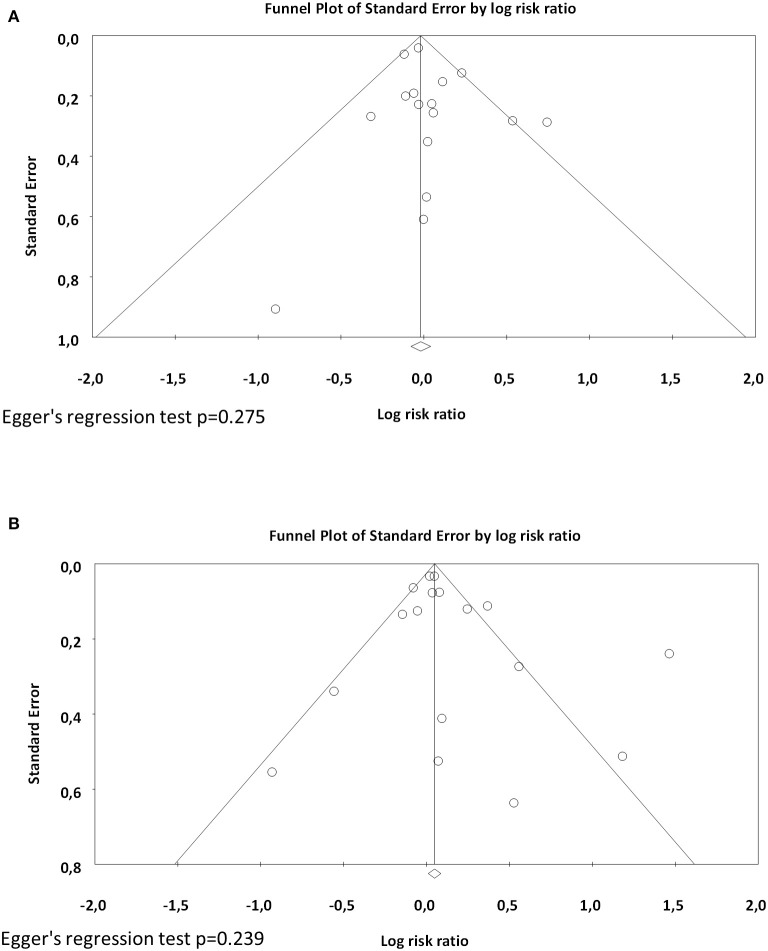
Funnel plots assessing publication bias for the association between maternal smoking during pregnancy and BPD **(A)** defined as oxygen requirement on postnatal day 28 (BPD28), and **(B)** defined as oxygen requirement at the postmenstrual age of 36 weeks (BPD36).

Sixteen studies reported data on BPD28 (15, 25–39) and meta-analysis could not find a significant association between maternal smoking and this outcome (RR 1.021, 95% CI 0.924–1.129, *p* = 0.681) (Figure 2). In contrast, the meta-analysis of the 17 studies that reported data on BPD36 (7, 8, 16, 39, 41–53) showed a significant association of maternal smoking with this outcome (RR 1.126, 95% CI 1.008–1.259, *p* = 0.036) (Figure 3). According to the GRADE methodology, the quality of the evidence on the association between maternal smoking and BPD36 was rated as low because of moderate RoB (see above) and low magnitude of the association (20). Only three studies reported on severe BPD (26, 39, 40). As shown in Figure 4, meta-analysis could not find a significant association between maternal smoking and severe BPD (RR 1.143, 95% CI 0.528–2.478, *p* = 0.734). Neither visual inspection of funnel plots (Figure 5) nor Egger's test (BPD28: *p* = 0.275; BPD36: *p* = 0.239) suggested publication or selection bias. There were insufficient studies with the definition of severe BPD to evaluate publication bias.

### Analysis of Heterogeneity

Neither subgroup-analysis (Supplementary Table 2) nor meta-regression (Supplementary Table 3) could demonstrate a significant effect of GA, BW, rate of maternal smoking in the total group, design of the study (cohort of case-control), sample size of the study (>100 infants), or continent (Europe or America) on the effect size of the association between maternal smoking and BPD.

## Discussion

To the best of our knowledge, this is the first systematic review and meta-analysis investigating the relationship between maternal smoking in pregnancy and BPD. Meta-analysis demonstrated a significant association between exposure to maternal smoking during pregnancy and risk of developing moderate/severe BPD (BPD36). In contrast, meta-analysis could not demonstrate a significant association between maternal smoking and all BPD (BPD28) or severe BPD. Our analysis is limited by the relative low number of studies and by the heterogeneity across studies. Studies on exposure to maternal smoking during pregnancy are by definition observational and, consequently, susceptible to confounding by unmeasured differences between the exposed and unexposed populations (20). In addition, information on maternal smoking was self-reported and no data were available on second-hand smoking during pregnancy. This may have led to a misclassification of the exposure (20). Therefore, according to the GRADE methodology, the quality of the evidence on the association between maternal smoking and moderate/severe BPD should be rated as low because of RoB and low magnitude of the association (20). Nevertheless, despite the methodological limitations, our data suggest that exposure to tobacco smoke during pregnancy may impair normal pulmonary growth and development, rendering preterm infants more susceptible to developing BPD.

Bias relating to exposure assessment is a major source of systematic error in studies of environmental exposures (20). Self-reported smoking is an inaccurate method of identifying smokers, with studies suggesting up to a 25% of pregnant smokers are missed when self-reporting is relied on (54). Social desirability bias is defined as “systematic error in self-report measures resulting from the desire of respondents to avoid embarrassment and project a favorable image to others” (55). Pregnant women may underreport smoking because their susceptibility to social messages of acceptability or disapproval in reference to “what a good mother does to protect her baby” (56, 57). Therefore, it is possible that an unknown percentage of infants of the “non-smoking” group had been actually exposed to maternal smoking. In addition, exposure to second-hand smoke in the non-smoker group may have masked the effects of smoking during pregnancy. It is estimated that more than a third of non-smoking women worldwide are exposed to second-hand smoke (58). Although exposure to second-hand smoke during pregnancy is generally less strongly associated with adverse health outcomes in the offspring than is maternal active smoking, it has been linked to conditions such as low birth weight or childhood asthma (11, 59, 60).

As discussed elsewhere (9, 61), meta-analyses of observational studies on BPD have to face the problem of the heterogeneity of BPD definition (1, 2, 62). The seminal description of BPD is credited to Northway et al. and dates back to 1968 (63). These authors described a picture of severe pulmonary damage as a result of mechanical ventilation and oxygen toxicity at a time when mortality related to neonatal respiratory distress syndrome was very high (1–3, 62). With the increased survival of very preterm infants, the clinical definition for BPD was adopted as “those infants requiring supplemental oxygen on postnatal day 28” (1, 3). Further refinement of the definition proposed the criterion of oxygen use at 36 weeks of PMA (64), and 12 years later, a division into three categories was proposed (18): (1) Mild BPD, need for supplementary oxygen or respiratory support at the postnatal age of 28 days but not at 36 weeks PMA; (2) Moderate BPD, need for 22–29% oxygen at 36 weeks PMA; and (3) Severe BPD, need for more than 30% oxygen and/or positive pressure at 36 weeks PMA (18, 65). Most of the studies included in our meta-analysis used the 36 weeks of PMA criterion to define BPD. Therefore, these studies combined moderate BPD with severe BPD. The main limitation of this combined outcome is that cannot isolate the group of infants with more severe BPD who remain dependent on mechanical ventilation and have a greater risk of severe complications such as pulmonary hypertension or alterations in growth and neurodevelopment (6). Despite our extensive search strategy, we only found three studies providing data on severe BPD. Meta-analysis could not demonstrate a significant association between smoking during pregnancy and risk of severe BPD, but the limited number of studies was an important limitation of this sub-analysis. Finally, another limitation of the current definitions and categorization of BPD is that not considering those infants who die of their respiratory failure before 36 weeks PMA (3). Very recently, Higgins et al. proposed a new classification of BPD that also included (as grade IIIA) this form of lethal BPD (3). Although such a refined definition is clinically reasonable, it will make future meta-analysis more challenging since change in disease definitions will affect the number of clinical studies that can be pooled for analysis.

That prenatal exposure to tobacco smoke increases the risk of BPD is a biologically plausible hypothesis. There is strong evidence from animal studies on the adverse impact of antenatal smoke exposure on lung development (10, 11). The effects of nicotine may occur very early, affecting embryonic stem cell differentiation (66). Intrauterine smoke exposure disrupts lung development in mice, resulting in a pathological picture similar to BPD (67). As reviewed by Gibbs et al., perinatal nicotine exposure in animal models induced alveolar hypoplasia, impaired lung branching morphogenesis, increased smooth muscle volume in distal bronchi, increased airway reactivity, and induced early immune dysregulation (10). In addition, nicotine-mediated reductions in placental blood flow and fetal oxygen and nutrient supply may play a role in the alteration of pulmonary growth and maturation and subsequent development of respiratory diseases (10, 11) Besides nicotine, other components of tobacco smoke may be detrimental to the developing lung (10, 11). Finally, tobacco smoke-related modification of the epigenome may have longitudinal effects on the patterns of DNA methylation and may alter the risk of lung disease in a transgenerational way (10, 11).

Risk of BPD is inversely correlated with GA and maternal smoking is a well-known risk factor for preterm birth. Therefore, an important part of the role of maternal smoking on the pathogenesis of BPD would be related to its role as a trigger for preterm birth. The etiopathogenesis of very (GA 28–32 weeks) and extremely (GA <28 weeks) preterm birth has been divided into two main categories: intrauterine infection/inflammation and placental vascular dysfunction. The first category includes pathological conditions such as chorioamnionitis, preterm labor, premature rupture of membranes, placental abruption, and cervical insufficiency. The second category is associated with gestational hypertensive disorders and the entity identified as fetal indication/fetal growth restriction (68–70). Smoking during pregnancy may interfere with both etiopathogenic mechanisms of preterm birth. Maternal smoking has been associated with increased risk of preterm labor, and premature rupture of membranes (71). In addition, as tobacco smoke seem to influence all aspects of the immune system, smoking may increase the risk of developing intrauterine infections, as well as non-genital maternal infections (71, 72). Moreover, maternal periodontal disease, which is more common among smokers compared with non-smokers (73), has been signaled as significant risk factor for chorioamnionitis and preterm delivery (74–76). On the other hand, maternal smoking induced changes in vascular histopathology, altered mechanical properties, and increased vasoconstrictive response in placental vessels (77, 78).

Exposure to tobacco smoke is associated with respiratory complications beyond BPD and the detrimental effects of smoking during pregnancy may also cumulate with smoking during lactation and with second-hand smoking exposure at home (11, 79). Maternal exposure to first- or second-hand tobacco smoke and prematurity appear to have a joint effect on respiratory complications, such as recurrent wheezing, or respiratory infections, among infants born very preterm (7, 11, 80). Exposure to second-hand smoke at home was associated with increased need for inhaled corticosteroids in children with BPD and with wheezing and need for acute care for respiratory problems in former very preterm infants (48, 81, 82). Therefore, a smoke-free environment is of critical importance for (very) preterm infants after discharge from the hospital.

In summary, the present meta-analysis confirmed the findings of previous individual studies on the association between maternal smoking during pregnancy and risk of BPD. Therefore, our results highlight the detrimental effects of tobacco smoking and reinforce the hypothesis of the involvement of prenatal insults in the etiopathogenesis of BPD. As assessed by Been and Millet, “children have the right to grow up in a smoke-free world” (12). Smoke-free legislation has the potential to reduce the substantive disease burden associated with tobacco-smoke exposure (13, 14). In addition, pregnancy and/or birth of a preterm baby should be considered as a major “teachable moment” to promote a healthy, smoke-free lifestyle for the whole family (11).

## Data Availability Statement

The datasets generated and analyzed for this study will be made available by the authors, without undue reservation, to any qualified researcher.

## Author Contributions

GG-L carried out the search and selected studies for inclusion, carried out data collection, carried out statistical analyses, assessed methodological quality, contributed to interpretation of results, helped draft the initial manuscript, and reviewed and revised the manuscript. EW-K carried out and supervised data collection, contributed to statistical analyses an interpretation of results, and reviewed and revised the manuscript. EV-M contributed to data collection, contributed to statistical analyses an interpretation of results, and reviewed and revised the manuscript. MH contributed to the search and inclusion of studies, contributed to interpretation of results, and reviewed and revised the manuscript. MK contributed to data collection, contributed to interpretation of results, and reviewed and revised the manuscript. BK contributed to interpretation of results and reviewed and revised the manuscript. EV conceptualized and designed the study, carried out the search and selected studies for inclusion, supervised data collection, assessed methodological quality, contributed to statistical analyses and interpretation of results, drafted the initial manuscript, and reviewed and revised the manuscript.

## Conflict of Interest

The authors declare that the research was conducted in the absence of any commercial or financial relationships that could be construed as a potential conflict of interest.
