# Supplementary material for: Tobacco Smoking During Pregnancy Is Associated With Increased Risk of Moderate/Severe Bronchopulmonary Dysplasia: A Systematic Review and Meta-Analysis
Source: Front Pediatr. 2020 Apr 28;8:160. doi: 10.3389/fped.2020.00160 (PMC7198744; doi:10.3389/fped.2020.00160)
Supplement: Supplementary file 1 [file Data_Sheet_1.PDF]

Supplementary Material

Tobacco smoking during pregnancy is associated with increased risk of moderate/severe bronchopulmonary dysplasia: A systematic review and meta-analysis

Gema E. González-Luis[1], Elke van Westering-Kroon[2], Eduardo Villamor-Martinez[2], Maurice J. Huizing[2], Mohammed A. Kilani[2], Boris W. Kramer[2], Eduardo Villamor[2*]

[1]Department of Pediatrics, Hospital Universitario Materno-Infantil de Canarias, Las Palmas de Gran Canaria, 35016, Spain

[2]Department of Pediatrics, Maastricht University Medical Center (MUMC+), School for Oncology and Developmental Biology (GROW), 6202AZ Maastricht, the Netherlands.

1 Supplementary Figures and Tables

Supplementary Table 1. Characteristics of all included studies

First author, Year	Country	Prospective /Retrospective	Cohort /Case-control	Perspective	Total infants (centers)	Mean or *median* GA	Mean or *median* BW	Definition BPD
**Alvarez-Fuente, 2019 [1]**	Spain	P	Cohort	BPD	47 (5)	*26*	*871*	BPD36
**Antonucci, 2004 [2]**	Italy	R	Cohort	BPD	277 (1)	30,1	1148	BPD28
**Baker, 2012 [3]**	USA	P	Cohort	BPD	62 (1)	31,6	1681	BPD36
**Berggren, 2010 [4]**	Sweden	R	Case-control	BPD	60 (1)	*28,5*	*1238*	BPD28<br>BPD36<br>BPDSevere
**Bose, 2009 [5]**	USA	R	Cohort	BPD	1209 (14)	25,6	834	BPD36
**Cazzato, 2013 [6]**	Italy	R	Case-control	BPD	48 (1)	28	1050	BPD28
**Demirel, 2009 [7]**	Turkey	R	Cohort	BPD	106 (1)	29,5	1218	BPD28
**Dietze, 2016 [8]**	USA	P	Cohort	BPD	25 (1)	29	1192	BPD28
**Eriksson, 2014 [9]**	Sweden	R	Cohort	BPD	96780 (Network)	-	-	BPD28
**Eriksson, 2015 [10]**	Sweden	R	Cohort	BPD	1880 (Network)	25,8	941	BPD36

First author, Year	Country	Prospective /Retrospective	Cohort /Case-control	Perspective	Total infants (centers)	Mean or *median* GA	Mean or *median* BW	Definition BPD
**Fujioka, 2014 [11]**	Japan	R	Case-control	BPD	95 (1)	*27,9*	*950*	BPD36
**Gage, 2015 [12]**	USA	R	Cohort	BPD	21944 (Network)	-	1383	BPD28
**Garcia-Morales, 2017 [13]**	Mexico	R	Cohort	BPD	57 (1)	*32*	*1400*	BPD28
**Gough, 2014 [14]**	UK	R	Case-control	BPD	129 (1)	29,1	1092	BPD28
**Hansen, 2010 [15]**	USA	P	Cohort	BPD	107 (1)	*29*	*1370*	BPD36
**Hennessy, 2008 [16]**	UK, Irland	P	Case-control	BPD	236 276	25	*743*	BPD36
**Isayama, 2015 [17]**	Canada	R	Cohort	Smoking	26646 (Network)	29,9	1313	BPD36
**Jeliazkova, 2012 [18]**	USA	R	Cohort	Smoking	1656 (1)	31	1682	BPD28
**Kennedy, 2000 [19]**	Australia	P	Case-control	BPD	99 (1)	29,6	1160	BPD28
**Korhonen, 2015 [20]**	Finland	R	Case-control	BPD	40 (1)	27,7	1052	BPD36
**Landry, 2016 [21]**	Canada	R	Cohort	BPD	88 (Network)	30,6	1730	BPD28
**Lodha, 2014 [22]**	Canada	R	Cohort	BPD	1030 (1)	*27,4*	*936*	BPD36
**Marom, 2016 [23]**	Israel	R	Case-control	BPD	78 (1)	27,2	937	BPD28
**Martinez, 2015 [24]**	France	R	Case-control	BPD	262 (Network)	30	1000	BPD28
**Morrow, 2017 [25]**	USA	P	Cohort	BPD	587 (Network)	*27*	*930*	BPD36
**Newman, 2011 [26]**	USA	P	Case-control	BPD	156 (1)	28,6	1177	BPD28 BPDSevere
**Norman, 2010 [27]**	Sweden	R	Cohort	BPD	497 (Network)	*24,9*	*770*	BPDSevere
**Praprotnik, 2015 [28]**	Slovenia	P	Case-control	BPD	56 (1)	27	1016	BPD36
**Sanchez-Solis, 2012 [29]**	Spain	P	Case-control	BPD	75 (1)	27,9	-	BPD28
**Soliman, 2017 [30]**	Canada	P	Cohort	BPD	319 (1)	*29*	*1206*	BPD36
**Spiegler, 2013 [31]**	Germany	R	Cohort	Smoking	2437 (40)	*28,9*	*1105*	BPD36

First author, Year	Country	Prospective /Retrospective	Cohort /Case-control	Perspective	Total infants (centers)	Mean or *median* GA	Mean or *median* BW	Definition BPD
**Torchin, 2016 [32]**	France	P	Cohort	BPD	2111 (Network)	*29,7*	*1266*	BPD36
**Yusuf, 2018 [33]**	Canada	R	Cohort	Smoking	12307 (Network)	26,5	884	BPD36

GA: gestational age; BW: birth weight; BPD: bronchopulmonary dysplasia; BPD28: bronchopulmonary dysplasia defined as oxygen dependency at 28 days of life; BPD36: bronchopulmonary dysplasia defined as oxygen dependency at 36 weeks post-menstrual age; Perspective BPD: studies that reported risk factors for BPD including maternal smoking; Perspective Smoking: studies that reported outcomes of maternal smoking.

References Supplementary Table 1

1. Alvarez-Fuente, M., et al., *Exploring clinical, echocardiographic and molecular biomarkers to predict bronchopulmonary dysplasia.* PLoS One, 2019. **14**(3): p. e0213210.
2. Antonucci, R., et al., *Intrauterine smoke exposure: a new risk factor for bronchopulmonary dysplasia?* J Perinat Med, 2004. **32**(3): p. 272-7.
3. Baker, C.D., et al., *Cord blood angiogenic progenitor cells are decreased in bronchopulmonary dysplasia.* Eur Respir J, 2012. **40**(6): p. 1516-22.
4. Broström, E.B., et al., *Obstructive lung disease in children with mild to severe BPD.* Respir Med, 2010. **104**(3): p. 362-70.
5. Bose, C., et al., *Fetal growth restriction and chronic lung disease among infants born before the 28th week of gestation.* Pediatrics, 2009. **124**(3): p. e450-8.
6. Cazzato, S., et al., *Lung function outcome at school age in very low birth weight children.* Pediatr Pulmonol, 2013. **48**(8): p. 830-7.
7. Demirel, N., A.Y. Bas, and A. Zenciroglu, *Bronchopulmonary dysplasia in very low birth weight infants.* Indian J Pediatr, 2009. **76**(7): p. 695-8.
8. Dietze, T.R., F.F. Rose, and T.A. Moore, *Maternal variables associated with physiologic stress and perinatal complications in preterm infants.* J Neonatal Perinatal Med, 2016. **9**(3): p. 271-7.
9. Eriksson, L., et al., *Prenatal inflammatory risk factors for development of bronchopulmonary dysplasia.* Pediatr Pulmonol, 2014. **49**(7): p. 665-72.
10. Eriksson, L., et al., *Perinatal conditions related to growth restriction and inflammation are associated with an increased risk of bronchopulmonary dysplasia.* Acta Paediatr, 2015. **104**(3): p. 259-63.
11. Fujioka, K., et al., *Association of a vascular endothelial growth factor polymorphism with the development of bronchopulmonary dysplasia in Japanese premature newborns.* Sci Rep, 2014. **4**: p. 4459.
12. Gage, S., et al., *Maternal Asthma, Preterm Birth, and Risk of Bronchopulmonary Dysplasia*, in *J Pediatr*. 2015, 2015 Elsevier Inc: United States. p. 875-880 e1.
13. García-Morales, E., et al., *Perfil epidemiológico de prematuros con displasia broncopulmonar en tercer nivel de atención.* Revista Médica MD www.revistamedicamd.com, 2017. **8**(4): p. 5.
14. Gough, A., et al., *Impaired lung function and health status in adult survivors of bronchopulmonary dysplasia.* Eur Respir J, 2014. **43**(3): p. 808-16.
15. Hansen, A.R., et al., *Maternal preeclampsia predicts the development of bronchopulmonary dysplasia.* J Pediatr, 2010. **156**(4): p. 532-6.
16. Hennessy, E.M., et al., *Respiratory health in pre-school and school age children following extremely preterm birth.* Arch Dis Child, 2008. **93**(12): p. 1037-43.

17. Isayama, T., et al., *Adverse Impact of Maternal Cigarette Smoking on Preterm Infants: A Population-Based Cohort Study.* Am J Perinatol, 2015. **32**(12): p. 1105-11.
18. Jeliazkova, Z., et al., *Effects of prenatal exposure to cigarette smoke on use of xanthine and pneumogram evaluation at discharge in premature infants.* J Matern Fetal Neonatal Med, 2012. **25**(6): p. 766-9.
19. Kennedy, J.D., et al., *Effects of birthweight and oxygen supplementation on lung function in late childhood in children of very low birth weight.* Pediatr Pulmonol, 2000. **30**(1): p. 32-40.
20. Korhonen, P.H., et al., *Inflammatory activity at school age in very low birth weight bronchopulmonary dysplasia survivors.* Pediatr Pulmonol, 2015. **50**(7): p. 683-90.
21. Landry, J.S., et al., *Lung Function and Bronchial Hyperresponsiveness in Adults Born Prematurely. A Cohort Study.* Ann Am Thorac Soc, 2016. **13**(1): p. 17-24.
22. Lodha, A., et al., *Need for supplemental oxygen at discharge in infants with bronchopulmonary dysplasia is not associated with worse neurodevelopmental outcomes at 3 years corrected age.* PLoS One, 2014. **9**(3): p. e90843.
23. Marom, R., et al., *Absolute nucleated red blood cells counts do not predict the development of bronchopulmonary dysplasia.* J Matern Fetal Neonatal Med, 2016. **29**(10): p. 1603-6.
24. Martinez, S., et al., *Tobacco smoke in infants with bronchopulmonary dysplasia.* Eur J Pediatr, 2015. **174**(7): p. 943-8.
25. Morrow, L.A., et al., *Antenatal Determinants of Bronchopulmonary Dysplasia and Late Respiratory Disease in Preterm Infants.* Am J Respir Crit Care Med, 2017. **196**(3): p. 364-374.
26. Newman, J.B., et al., *Neonatal respiratory dysfunction and neuropsychological performance at the preschool age: a study of very preterm infants with bronchopulmonary dysplasia.* Neuropsychology, 2011. **25**(5): p. 666-678.
27. Norman, M., et al., *Incidence of and risk factors for neonatal morbidity after active perinatal care: extremely preterm infants study in Sweden (EXPRESS).* Acta Paediatr, 2010. **99**(7): p. 978-92.
28. Praprotnik, M., et al., *Respiratory morbidity, lung function and fitness assessment after bronchopulmonary dysplasia.* J Perinatol, 2015. **35**(12): p. 1037-42.
29. Sanchez-Solis, M., et al., *Lung function among infants born preterm, with or without bronchopulmonary dysplasia.* Pediatr Pulmonol, 2012. **47**(7): p. 674-81.
30. Soliman, N., et al., *Preeclampsia and the Risk of Bronchopulmonary Dysplasia in Preterm Infants Less Than 32 Weeks' Gestation.* Am J Perinatol, 2017. **34**(6): p. 585-592.
31. Spiegler, J., et al., *Influence of smoking and alcohol during pregnancy on outcome of VLBW infants.* Z Geburtshilfe Neonatol, 2013. **217**(6): p. 215-9.
32. Torchin, H., et al., *Placental Complications and Bronchopulmonary Dysplasia: EPIPAGE-2 Cohort Study.* Pediatrics, 2016. **137**(3): p. e20152163.
33. Yusuf, K., et al., *Neonatal outcomes of extremely preterm infants exposed to maternal hypertension and cigarette smoking.* J Perinatol, 2018. **38**(8): p. 1051-1059.

Supplementary Table 2. Subgroup analyses.

BPD	Grouping variable	Subgroup	k	RR (95% CI)	p-value	Heterogeneity	
						I^2	p-value
BPD28	Continent	America	6	1.099 (0.884-1.367)	0.397	36%	0.167
		Europe	7	0.959 (0.794-1.159)	0.666	31%	0.192
	Study design	Cohort	7	1.124 (0.916-1.378)	0.263	61%	0.019
		Case-control	9	0.980 (0.809-1.186)	0.835	0%	0.600
	Sample size	>100	8	0.997 (0.883-1.125)	0.963	9%	0.357
		≤100	8	1.176 (0.914-1.515)	0.208	43%	0.092
BPD36	Continent	America	8	1.191 (0.987-1.438)	0.068	86%	<0.001
		Europe	8	1.146 (0.925-1.420)	0.214	48%	0.060
	Study design	Cohort	12	1.135 (0.973-1.325)	0.106	81%	<0.001
		Case-control	5	1.183 (0.871-1.607)	0.281	0%	0.026
	Sample size	>100	11	1.124 (0.972-1.301)	0.116	82%	<0.001
		≤100	6	1.281 (0.883-1.859)	0.193	54%	0.054

BPD28: bronchopulmonary dysplasia defined as oxygen dependency at 28 days of life; BPD36: bronchopulmonary dysplasia defined as oxygen dependency at 36 weeks post-menstrual age; k: number of studies included in analysis; RR: risk ratio; CI: confidence interval.

Supplementary Table 3. Meta-regression analyses

BPD definition	Co-variate	k	Coefficient	95% CI	p-value
BPD28	Mean/median GA (per week)	14	0.147	-0.042 to 0.336	0.127
	Mean/median BW (per 100g)	14	0.025	-0.056 to 0.106	0.544
	% smoking in total group	16	-0.119	-1.203 to 0.965	0.830
BPD36	Mean/median GA	17	-0.004	-0.113 to 0.105	0.938
	Mean/median BW (per 100g)	17	0.019	-0.078 to 0.116	0.698
	% smoking in total group	17	-0.103	-2.455 to 2.249	0.932

BPD28: bronchopulmonary dysplasia defined as oxygen dependency at 28 days of life; BPD36: bronchopulmonary dysplasia defined as oxygen dependency at 36 weeks post-menstrual age; k: number of studies included in analysis; RR: risk ratio; CI: confidence interval; GA: gestational age; BW: birth weight.

Search strategy

Full details of PubMED search

(("mothers"[MeSH Terms] OR "mothers"[tiab] OR "maternal"[tiab]) AND ("smoking"[MeSH Terms] OR "smoking"[tiab])) AND (("premature birth"[MeSH Terms] OR ("premature"[tiab] AND "birth"[tiab]) OR "premature birth"[tiab] OR ("preterm"[tiab] AND "birth"[tiab]) OR "preterm birth"[tiab]) OR ("bronchopulmonary dysplasia"[MeSH Terms] OR ("bronchopulmonary"[tiab] AND "dysplasia"[tiab]) OR ("chronic lung disease"[tiab]) OR "bronchopulmonary dysplasia"[tiab]) OR ("infant, low birth weight"[MeSH Terms] OR ("infant"[tiab] AND "low"[tiab] AND "birth"[tiab] AND "weight"[tiab]) OR "low birth weight infant"[tiab] OR ("low"[tiab] AND "birth"[tiab] AND "weight"[tiab]) OR "low birth weight"[tiab]) OR ("infant, very low birth weight"[MeSH Terms] OR ("infant"[tiab] AND "low"[tiab] AND "birth"[tiab] AND "weight"[tiab]) OR "very low birth weight infant"[tiab] OR ("low"[tiab] AND "birth"[tiab] AND "weight"[tiab]) OR "very low birth weight"[tiab]) OR prematurity[tiab]) AND (("cohort studies"[MeSH Terms] OR ("cohort"[tiab] AND "studies"[tiab]) OR "cohort studies"[tiab]) OR ("observational study"[Publication Type] OR "observational studies as topic"[MeSH Terms] OR "observational study"[tiab]) OR ("case-control studies"[MeSH Terms] OR ("case-control"[tiab] AND "studies"[tiab]) OR "case-control studies"[tiab] OR ("case"[tiab] AND "control"[tiab] AND "studies"[tiab]) OR "case control studies"[tiab]))

OR

(("risk factors"[MeSH Terms] OR ("risk"[tiab] AND "factors"[tiab]) OR "risk factors"[tiab]) AND ("bronchopulmonary dysplasia"[MeSH Terms] OR ("bronchopulmonary"[tiab] AND "dysplasia"[tiab]) OR ("chronic lung disease"[tiab]) OR "bronchopulmonary dysplasia"[tiab])) AND (("cohort studies"[MeSH Terms] OR ("cohort"[tiab] AND "studies"[tiab]) OR "cohort studies"[tiab]) OR ("observational study"[Publication Type] OR "observational studies as topic"[MeSH Terms] OR "observational study"[tiab]) OR ("case-control studies"[MeSH Terms] OR ("case-control"[tiab] AND "studies"[tiab]) OR "case-control studies"[tiab] OR ("case"[tiab] AND "control"[tiab] AND "studies"[tiab]) OR "case control studies"[tiab]))

Full details of Embase search

(maternal smoking/ AND (premature labor/ OR lung dysplasia/ OR low birth weight/ OR prematurity/))

OR

(risk factor/ AND lung dysplasia/ AND (cohort analysis/ OR observational study/ OR case control study/))
